# Improvement in Prediction of Coronary Heart Disease Risk over Conventional Risk Factors Using SNPs Identified in Genome-Wide Association Studies

**DOI:** 10.1371/journal.pone.0057310

**Published:** 2013-02-27

**Authors:** Jennifer L. Bolton, Marlene C. W. Stewart, James F. Wilson, Niall Anderson, Jackie F. Price

**Affiliations:** 1 Centre for Population Health Sciences, Medical School, Teviot Place, University of Edinburgh, Edinburgh, United Kingdom; 2 MRC Institute of Genetics and Molecular Medicine, University of Edinburgh, Western General Hospital, Edinburgh, United Kingdom; University of Texas School of Public Health, United States of America

## Abstract

**Objective:**

We examined whether a panel of SNPs, systematically selected from genome-wide association studies (GWAS), could improve risk prediction of coronary heart disease (CHD), over-and-above conventional risk factors. These SNPs have already demonstrated reproducible associations with CHD; here we examined their use in long-term risk prediction.

**Study Design and Setting:**

SNPs identified from meta-analyses of GWAS of CHD were tested in 840 men and women aged 55–75 from the Edinburgh Artery Study, a prospective, population-based study with 15 years of follow-up. Cox proportional hazards models were used to evaluate the addition of SNPs to conventional risk factors in prediction of CHD risk. CHD was classified as myocardial infarction (MI), coronary intervention (angioplasty, or coronary artery bypass surgery), angina and/or unspecified ischaemic heart disease as a cause of death; additional analyses were limited to MI or coronary intervention. Model performance was assessed by changes in discrimination and net reclassification improvement (NRI).

**Results:**

There were significant improvements with addition of 27 SNPs to conventional risk factors for prediction of CHD (NRI of 54%, *P*<0.001; C-index 0.671 to 0.740, *P* = 0.001), as well as MI or coronary intervention, (NRI of 44%, *P*<0.001; C-index 0.717 to 0.750, *P* = 0.256). ROC curves showed that addition of SNPs better improved discrimination when the sensitivity of conventional risk factors was low for prediction of MI or coronary intervention.

**Conclusion:**

There was significant improvement in risk prediction of CHD over 15 years when SNPs identified from GWAS were added to conventional risk factors. This effect may be particularly useful for identifying individuals with a low prognostic index who are in fact at increased risk of disease than indicated by conventional risk factors alone.

## Introduction

There has been much discussion of personalised medicine and the use of genetic risk scores for identifying people at increased risk for chronic diseases including coronary heart disease (CHD). The expectation is that such individuals might benefit from targeted interventions, thereby reducing their risk of developing disease. The Framingham risk score [1] is the most commonly used method of CHD risk prediction, and has been widely assessed for validity. However, the accuracy of this score differs between populations, commonly over-estimating risk in European countries [2], and overall accuracy is generally low for individuals not at the extremes of risk distributions. Alternative risk prediction models have been developed which incorporate a range of additional risk factors, such as biomarkers [3], socio-economic indicator, or family history [4], but these still have limited predictive power.

Family history is predictive of CHD after adjusting for other conventional risk factors [5], [6], and CHD is estimated to be approximately 40–50% heritable [7], [8]. Despite this, genetic information has so far generally not resulted in appreciable improvements in prediction over non-genetic risk factors, (apart from monogenic disease). This is likely due in part to the small effects exerted by individual single nucleotide polymorphisms (SNPs) relative to established risk factors; but the selection of SNPs for evaluation, and methods of inclusion in a predictive model, are also likely contributors. Previous genetic risk prediction models have often relied on candidate SNPs that have a known biological role in, or association with, CHD or atherosclerosis [9]. The publication of genome-wide association studies (GWAS) has provided another method for identification of SNPs, independent of known biological function, but based on statistical evidence of association. Models have often used genetic risk scores, basically a sum of the number of risk alleles, which do not take into account the individual effect sizes and assume independence of these alleles.

The primary aim of this analysis was to determine whether a systematically selected panel of SNPs, already found individually to be reproducibly associated with CHD through GWAS, could improve prediction of CHD over and above well established conventional risk factors, thereby contributing additional clinical utility. Since the majority of coronary events occur in individuals with Framingham based risk scores of less than 20% [10], the inclusion of genetic information has the potential to create a more personalised and accurate risk evaluation.

## Methods

### Study Population

Details of the Edinburgh Artery Study (EAS), have been published previously [11], [12]. In brief, the EAS enrolled 1592 men (809) and women (783) aged 54–75 years living in Edinburgh, Scotland. Recruitment used an age-stratified random sample from ten general practices, resulting in a geographical and socio-economic representation of the population of Edinburgh. Clinical examinations were held during 1987/8, and DNA samples were collected at a five year follow-up examination (attended by 1165 (73%) subjects). At time of genotyping for the current study (2009), DNA was available for 856 subjects, of which 840 were successfully genotyped (409 men, 431 women). Reasons for not having a DNA sample included refusal to provide a blood sample or allow genotyping at the 5-year examination, or insufficient sample remaining. Baseline characteristics of the full EAS population and the population used for the current analysis were very similar (Table 1).

**Table 1:** Comparison of baseline characteristics of the EAS population used in genetic risk prediction models and full study population.

	Study population (1592)	Genotyped population (840)
	Mean (95%CI)	Mean (95%CI)
Age at baseline	64.9 (64.6,65.1)	64.4 (64.0,64.8)
Body Mass Index	25.6 (25.4,25.8)	25.5 (25.3,25.8)
Systolic Blood Pressure	144 (143,146)	143 (142,145)
Diastolic Blood Pressure	77 (77,78)	77 (77,78)
Total Cholesterol	7.03 (6.97,7.10)	7.08 (6.99,7.02)
HDL Cholesterol	1.44 (1.42,1.46)	1.45 (1.42,1.50)
LDL Cholesterol	5.28 (5.22,5.34)	5.33 (5.25,5.40)
log(Triglycerides)	0.15 (0.14,0.16)	0.14 (0.13,0.20)
	**n (%)**	**n (%)**
Sex Male	809 (51)	409 (49)
Diabetes	288 (18.1)	136 (16.2)
Family History in parent	576 (36.2)	257 (38.0)
Current Smoker	404 (25.4)	182 (21.7)
Previous Smoker	582 (36.6)	315 (37.5)
Never Smoked	561 (35.2)	328 (39.0)

Data collection for identification and validation of coronary events at baseline and throughout follow-up included the WHO chest pain questionnaire, ECG (coded using Minnesota Classification Code), self-reported doctor diagnosis of disease, record linkage to hospital discharge data and death certificates, and scrutiny of general practitioner records [12]. Conventional risk factors measured at baseline included lipids and blood pressure. Complete follow-up was available until June 2003, a mean follow-up of 15 years.

The classification of CHD used in the current analyses was based on validated events and comprised of fatal or non-fatal myocardial infarction (MI), angioplasty, coronary artery bypass surgery, angina and/or unspecified ischaemic heart disease as a cause of death. To reduce the potential for mis-classification, further analyses were restricted to fatal or non-fatal MI or coronary intervention (angioplasty or coronary artery bypass surgery). Family history was also collected at baseline, but was limited to unconfirmed self-reports of MI or angina in a parent.

### Ethical Approvals

Ethical approval for the EAS was granted by the Lothian Health Board Medical Research Ethics Committee. Written informed consent was obtained from all participants.

### SNP Identification

Selection of SNPs used recent large scale meta-analyses of GWAS of CHD to identify SNPs that have demonstrated reproducible associations with CHD [13], [14]. This provided 36 SNPs, of which six were not available on Metabochip (rs10953541, rs1412444, rs17609940, rs216172, rs46522, rs964184) and no proxy was available; rs4977574 was replaced with rs133049 (r^2^ = 0.97, D’ = 1.0). Three SNPs were removed because they were in LD (r^2^>0.85) with other included SNPs (rs646776, rs1199338, rs12526453). Details of SNPs used in prediction models are presented in Table 2 (detailed in Table S1).

**Table 2:** SNPs identified from meta-analysis of GWAS of CHD used in risk prediction models.

SNP	Chr	Position (b37)	Gene(s)	Alleles	Minor allele	MAF
rs11206510	1	55,268,627	PCSK9	C/T	C	0.16
rs17114036	1	56,735,409	PPAP2B	A/G	G	0.11
rs599839	1	109,623,689	SORT1	A/G	G	0.28
rs17011666	1	220,865,588	MIA3	A/G	G	0.17
rs17465637	1	220,890,152	MIA3	A/C	A	0.27
rs6725887	2	203,454,130	WDR12	C/T	C	0.16
rs2306374	3	139,602,642	MRAS	C/T	C	0.18
rs1332844	6	12,996,990	PHACTR1	C/T	C	0.39
rs12190287	6	134,256,218	TCF21	C/G	G	0.40
rs3798220	6	160,881,127	LPA	C/T	C	0.00
rs11556924	7	129,450,732	ZC3HC1	C/T	T	0.39
rs1333049	9	22,115,503	CDKN2A,	C/G	C	0.46
rs579459	9	135,143,989	ABO	C/T	C	0.20
rs2505083	10	30,375,128	KIAA1462	C/T	C	0.43
rs1746048	10	44,095,830	CXCL12	C/T	T	0.15
rs12413409	10	104,709,086	CYP17A1, CNNM2, NT5C2	A/G	A	0.08
rs974819	11	103,165,777	PDGFD	C/T	T	0.22
rs3184504	12	110,368,991	SH2B3	C/T	T	0.45
rs4773144	13	109,758,713	COL4A1, COL4A2	A/G	G	0.42
rs2895811	14	99,203,695	HHIPL1	C/T	C	0.42
rs3825807	15	76,876,166	ADAMTS7	A/G	G	0.45
rs4380028	15	76,898,148	ADAMTS7-MORF4L1	C/T	T	0.41
rs12936587	17	17,484,447	RASD1, SMCR3, PEMT	A/G	G	0.47
rs1122608	19	11,024,601	LDLR	G/T	T	0.26
rs2228671	19	11,071,912	LDLR	C/T	T	0.11
rs9982601	21	34,520,998	MRPS6	C/T	T	0.21
rs7278204	21	34,543,235	SLC5A3-MRPS6-KCNE2	A/G	G	0.17

Additional SNPs for use in a secondary, exploratory analysis were selected based on nominal significance (*P*<1×10^−5^) in GWAS of CVD, significant associations with lipids in GWAS, and/or biological plausibility. This provided an additional 44 SNPs (detailed in Table S2) that were available and successfully genotyped in the study population, resulting in a total set of 74 SNPs for use in secondary analysis. This was a more subjectively selected and therefore potentially biased set of SNPs.

### Genotyping

Genotyping used the Illumina MetaboChip, from which the chosen SNPs were extracted. Quality control was carried out on the full MetaboChip results, 16 samples with call rates below 75% were excluded. Table S1 reports: call rates, mean genotypic call rate of 97.7% (range 85.5–99.5); Hardy Weinberg Equilibrium (HWE), one SNPs showed deviation from HWE (rs4773144); and minor allele frequencies (MAF), range 3–49%.

### Statistical Analysis

Statistical analysis used R version 2.14.0 [15], all p-values were two-sided. Prediction of coronary risk used multivariate adjusted Cox proportional hazards in the *survival* library [16], the assumption of proportional hazards was satisfied for all models. Conventional risk factors were based on the Framingham model [1], and included: sex, baseline age, systolic blood pressure, smoking (Yes/No), diabetes and/or glucose intolerance (Yes/No), and total cholesterol/HDL cholesterol. SNPs were added as covariates to the conventional risk factors, assuming an additive model. This was thought preferable to creation of a single genetic risk score as it allows more influential SNPs to exert more of an effect on the model, whereas a composite risk score assumes all SNPs have the same effect size. The derived ß coefficients were used to calculate prognostic indices, thereby creating weighted prediction models. Prognostic indices were converted to predicted probabilities as 1−S_0_(t)^exp(PI)^ [1].

Model performance was evaluated by C-indices, net reclassification indices (NRI), integrated discrimination improvement (IDI), and plotted ROC curves. ROC curves were plotted using the *ROCR* library [17], C-indices, NRI, and IDI used the *Hmisc* library [18]. The C-index used in survival analysis is analogous to area under the ROC curve used in logistic regression, simply it is a measure of the concordance in predicted and observed survival times between subjects [19]. NRI was based on event specific reclassification and used continuous measures rather than categories, which increases statistical power. NRI can be used to compare the clinical impact of different models, simply, it is a comparison of the proportion of subjects with disease who have appropriately increased risk scores with the new model, and the proportion of subjects without disease who have appropriately decreased risk scores with the new model [20]. IDI represents desired improvements in average sensitivity corrected for undesirable increases in 1-specificity, it therefore compared whether the new models improved sensitivity without affecting specificity, as described in Pencina *et al.* (2008). ROC curves are plots of 1-specificity vs sensitivity, allowing visualisation of changes in discrimination over different sensitivities.

All analyses used first incident events only, subjects with a diagnosis of prevalent CHD at baseline were excluded, as appropriate. Time to event was determined individually for both CHD and fatal or non-fatal MI or coronary intervention, based on appropriate diagnostic criteria. Since models based on different subjects could differ, risk prediction models that were compared contained identical population groups. Power was calculated using the *gap* library [21]. Though underpowered to detect significant associations for individual SNPs, it was hypothesised that a set of SNPs with high prior probability could jointly have a sufficiently large effect size. There was 80% power to detect an effect size of 1.5 with a minor allele frequency (MAF) of 30% in a multiplicative model, at 5% significance with a disease prevalence of 20%.

An exploratory method of selecting SNPs used regression trees in the *rpart* library [22]. To identify SNPs that were informative after conventional risk factors, the residuals of a model containing conventional risk factors were used as the dependent variable. This analysis included the full collection of 74 SNPs as potential covariates. Tree development used the Gini Index as the splitting rule, SNPs were treated as ordinal, and splitting was only considered as dominant or recessive models. Regression trees sequentially selected SNPs that best partitioned subjects into the appropriate group [23]; the sets of SNPs that were identified by the regression trees were then used to develop prediction models.

## Results

### Risk Prediction Using SNPs with Confirmed Associations with CHD

27 SNPs identified in meta-analysis of GWAS of CHD were successfully genotyped in the EAS population (Table 2), and results of prediction models are summarised in Table 3 (hazard ratios given in Tables S3 and S4). Addition of the 27 SNPs to conventional risk factors in prediction of CHD increased the C-index from 0.671 to 0.740 (*P* = 0.001) and NRI was 54% (95%CI 35–74; *P*<0.001). When restricted to fatal or non-fatal MI or coronary intervention the C-index increased from 0.717 to 0.750 (*P* = 0.256), and NRI was 44% (95%CI 20–67; *P*<0.001). The results were almost identical when family history of CHD was also included in the models.

**Table 3:** Incidence, Discrimination, and Calibration Estimates of Models Using Conventional Risk Factors* and GWAS or Regression Tree SNPs in the EAS.

	Concordance		R^2^	C-index	NRI (95% CI)	NRI event/nonevent		IDI (95% CI)
**SNPs identified through GWAS of CHD**								
**CHD (n = 508, 131 incident events)**								
Conventional risk factors		0.658	0.081	0.671				
Conventional risk factors & SNPs		0.712	0.137	0.740	54.4 (34.5,74.3)		17.6/36.9	0.04 (0.02,0.06)
Conventional risk factors & Family history		0.658	0.082	0.671				
Conventional risk factors, Family history & SNPs		0.712	0.138	0.741	54.4 (34.5,74.3)		17.6/36.9	0.04 (0.02,0.06)
**Fatal or non-fatal MI or coronary intervention (n = 590, 81 incident events)**								
Conventional risk factors		0.701	0.062	0.717				
Conventional risk factors & SNPs		0.731	0.106	0.750	43.5 (20.1,67.0)		11.1/32.4	0.05 (0.02,0.08)
Conventional risk factors & Family history		0.702	0.063	0.718				
Conventional risk factors, Family history & SNPs		0.734	0.107	0.753	42.7 (19.3,66.2)		11.1/31.6	0.05 (0.02,0.07)
**SNPs identified through Regression Trees**								
**CHD (n = 663, 180 incident events)**								
Conventional risk factors		0.652	0.077	0.686				
Conventional risk factors & SNPs		0.686	0.124	0.709	41.5 (24.6,58.4)		21.5/20.0	0.04 (0.02,0.05)
**Fatal or non-fatal MI or coronary intervention (n = 768, 107incident events)**								
Conventional risk factors		0.679	0.050	0.694				
Conventional risk factors & SNPs		0.704	0.077	0.718	42.9 (22.5,63.3)		14.0/28.9	0.03 (0.01,0.04)

* Conventional risk factors = Age, Sex, SBP, Total Cholesterol/HDL Cholesterol, Diabetes and/or glucose intolerance, Smoking.

Each analysis used only subjects without a diagnosis at baseline, as appropriate to investigate incident events, and with full genotypic data for included SNPs.

**Figure 1:**
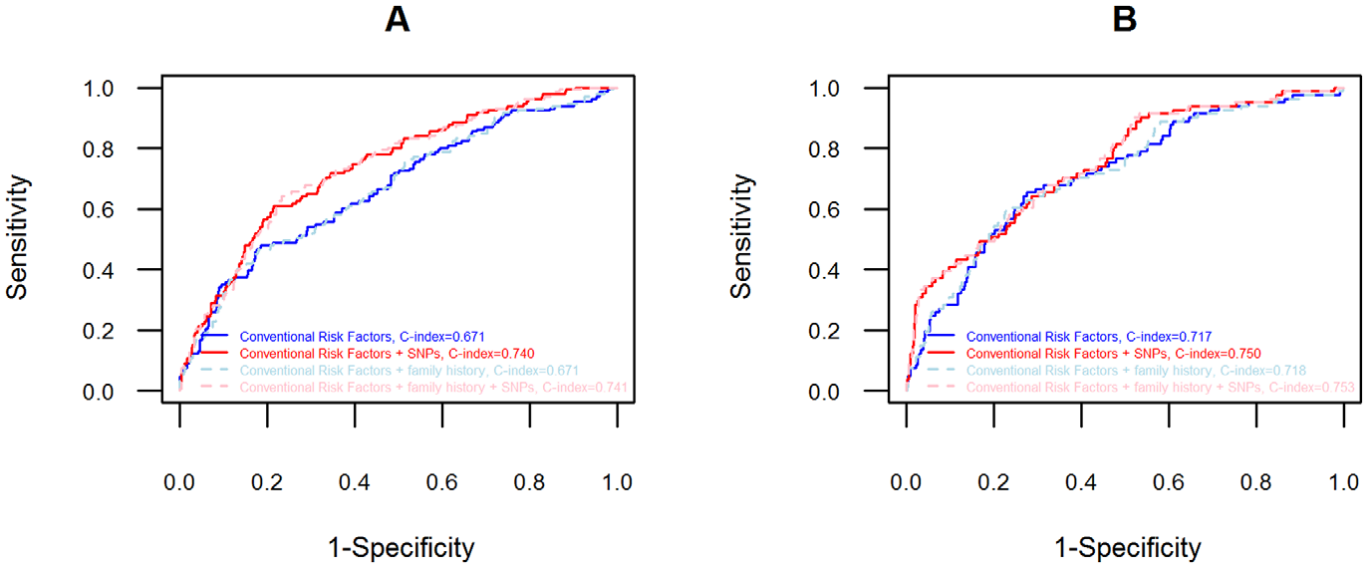
ROC curves of prediction of coronary heart disease when GWAS significant SNPs were added to conventional risk factors. A: ROC curves for CHD, comprised of fatal or non-fatal MI, angioplasty, coronary artery bypass surgery, angina and/or unspecified ischaemic heart disease as a cause of death; B: ROC curves for diagnoses limited to fatal or non-fatal MI or coronary intervention (angioplasty or coronary artery bypass surgery).

Plotted ROC curves (Figure 1) showed that addition of SNPs improved prediction over much of the curve for CHD, however for fatal or non-fatal MI or coronary intervention the models performed differently at different sensitivities when SNPs were added; here the addition of SNPs better improved discrimination when the sensitivity of conventional risk factors was lower, translating to improved identification of an individual with a low prognostic index in fact at increased risk of an event. This was mirrored in density plots, in which a second distribution of higher risk scores for subjects with events emerged upon addition of SNPs (Figure S1). Addition of SNPs to conventional risk factors moved 10 subjects to predicted risk ≥20%, and increased the OR of having any CHD given a ≥20% predicted risk increased from 3.86 (95%CI 2.52,5.93) to 5.42 (95%CI 3.54,8.38). When restricted to fatal or non-fatal MI or coronary intervention, 16 subjects moved to predicted risk ≥20%., and the odds ratio of having an event given a ≥20% predicted risk increased from 4.42 (95%CI 1.78,10.46) to 12.18 (95%CI 6.30,24.03).Reclassification tables are presented in Table S5.

### Risk Prediction Using SNPs Identified from Regression Trees

The use of regression trees to identify SNPs that explained the remaining variance after consideration of conventional risk factors was a secondary, exploratory approach to developing prediction models. Of the potential 74 SNPs, seven SNPs were found to explain some of the remaining risk of CHD: rs1122608 (*SMARCA1*), rs3798220 (*LPA*), rs780094 (*GCKR*), rs1332844 (*PHACTR1*), rs11668477 (*LDLR*), rs3184504 (*SH2B3*), rs2505083 (*KIAA1462*). When done for fatal or non-fatal MI or coronary intervention, the list of nine predictive SNPs differed: rs780094 (*GCKR*), rs17011666 (*MIA3*), rs11556924 (*ZC3HC1*), rs3798220 (*LPA*), rs4939883 (*LIPG*), rs12413409 (*CNNM2*), rs17145738 (*TBL2/MLXIPL*), rs174570 (*FADS1/2*), rs173539 (*CETP)*. Regression trees are shown in Figure S2, model results in Table 3, and ROC curves in Figure S3. Addition of regression tree SNPs to conventional risk factors increased the C-index for prediction of CHD from 0.668 to 0.709, (*P* = 0.027), had a NRI of 42% (95%CI 25,58; *P*<0.001), and moved six subjects with CHD to predicted risk ≥20%. The SNPs predictive of fatal or non-fatal MI or coronary intervention increased the C-index from 0.694 to 0.718 (*P* = 0.463), had a NRI of 43% (95%CI 22,63; *P*<0.001), and moved 15 subjects to predicted risk ≥20%.

## Discussion

In this prospective, population-based cohort of men and women from Edinburgh, Scotland, a systematically-selected set of SNPs improved prediction of CHD over 15 years, over-and-above conventional risk factors. A total of 27 SNPs that were significantly associated with CHD, when added to the Framingham-based conventional risk factors of age, sex, SBP, total cholesterol/HDL cholesterol, diabetes and/or glucose intolerance, and smoking, improved prediction as indicated by significant improvements in NRI and C-indices. NRI were used to evaluate the clinical impact of addition of SNPs. Given that an estimated 15–20% of MI occur in individuals considered as lower risk based on conventional risk factors [24], the ability of this genetic model to identify such subjects and increase their predicted risk indicates potential clinical utility. The highest risk category of at least 20% CHD risk was of interest as individuals in this category are often considered suitable for clinical intervention, and the risk of mis-classification is decreased [25]. The appropriate reclassification of subjects to ≥20% predicted risk on addition of SNPs to conventional risk factors suggests that such a model could affect treatment decisions for a number of individuals.

Regression trees were used to evaluate whether a smaller collection of SNPs was sufficient to improve prediction, to account for the possibility that not all SNPs contribute to prediction. This allowed for selection of additional SNPs as it was not expected that GWAS would have sufficient power to identify all associated and/or predictive SNPs. Though regression trees are prone to over fitting, they were an exploratory method to limit the number of SNPs included in the models. They also provided branching patterns that may show that an effect at one SNP may only occur in the presence of another SNP. This would indicate that only SNPs with independent effects should be included, in order to get more accurate population based risk associated with the SNP.

Previous studies that added candidate SNPs to conventional risk factors, using either genetic risk scores (a count of the number or risk alleles) or weighting of SNPs, have generally not significantly improved model discrimination as measured by C-index. They have however indicated through NRI [26] and/or increased hazard ratios that SNPs could improve risk prediction [27], [28], [29], [30], [31]; with significant associations reported between incident CHD and genetic risk scores [26], [27], [28]. Humphries *et al.* (2007) [32] found a significant improvement in C-index in the Northwick Park Heart Study II (*P*<0.001), which was further improved after inclusion of an interaction with smoking (*P* = 0.01).

More recently there have been other studies that used GWAS-identified SNPs in prospective cohorts; these contained many but not all of the GWAS SNPs used in the present analyses. Paynter *et al*. (2010) [6] assessed the predictive ability of adding genetic risk scores to conventional risk factors for prediction of any CVD (MI, stroke, arterial revascularization, and cardiovascular death) in a large cohort, and found no improvement in discrimination or reclassification. Additionally the investigators found that the genetic risk score alone was not associated with risk of CVD; this may be due to the use of a broader phenotype. Davies *et al*. (2010) [33] assessed the predictive utility for CHD and found a significant improvement in the C-index when SNPs were added to conventional risk scores, from 0.801 to 0.809, (*P* = 0.0073). They additionally found that weighting SNPs led to models that performed better than unweighted models. Ripatti et al. (2010) [34] also reported an association between incident CHD and genetic risk score after adjusting for conventional risk factors, however this was observed through improvements in IDI and NRI, and did not lead to significant changes in C-index. They also reported that though family history was associated with increased risk of CVD, adjustment for family history did not change the risk estimates of the genetic risk score.

The use of a genetic risk score results in equal weighting of all SNPs, possibly missing relevant information on the relative effects of each SNP within the model [32]. Also, in the development of a model in which covariates are not unrelated, the ß coefficients need to be adjusted to account for the impact covariates have on each other to prevent distortion of the model. ROC curves measure discrimination but are ‘insensitive to change’ [19], [35], however as our curves showed, the changes in risk prediction did not always change consistently over the full range of sensitivities, a large change in one portion of the curve may be clinically relevant but not represented in summary measures. The clinical value was demonstrated by the increased NRI, and subsequent increased odds of subjects with CHD to have predicted risk ≥20%, showing that addition of GWAS SNPs can have clinical applicability.

There were a number of strengths and weaknesses of the current study. A strength of the EAS population was the long follow-up of 15 years, and the prospective method that included regular contact with study participants and general practitioners, as well as use of hospital discharge records and death certificates. This enabled confirmation of reported events, providing accurate phenotypic data and minimising misclassification bias; as well as detailed and accurate records for subjects that died during follow-up, thereby removing survivor bias. Here we found that genetic data was more informative than self-reported family history of CHD. This was possibly due to the difficulty in collecting accurate reports of family history in epidemiological studies, which would also exist clinically and therefore not result in accurate risk prediction.

As the cohort was recruited from Edinburgh only and was primarily white, the risk of population stratification was low. However, the EAS study population was small for a genetic study. There may also have been temporal trends that affected CHD risk and consequently risk prediction, such as smoking habits and primary prevention of CHD. At baseline, medications for CHD risk factors were not as commonly used as recently, and during follow-up a considerable portion of the population were prescribed anti-hypertensive, lipid lowering, and/or diabetes treatments. With such a long follow-up this may have been a confounder that was not accounted for.

This is not a definitive list of predictive SNPs. Further analysis of GWAS and fine mapping is necessary to identify causal SNPs that will be more accurate in risk prediction. There remains the possibility that some of the GWAS significant SNPs did not contribute to risk prediction. It is also likely that there are gene-environment and gene-gene interactions that were not accounted for, for example Humphries *et al*. (2007) found an interaction with smoking [32], and *HMGCR* genotypes may affect lipid lowering responses to statins [36]. Though use of GWAS results removed sources of bias associated with the inclusion of candidate gene study results, there remain problems specifically associated with GWAS, such as poor representation of low MAF SNPs, that debatably have larger effect sizes [37]. However, we have shown that use of a systematically selected panel of SNPs can significantly improve prediction of CHD risk over-and-above conventional risk factors, indicating that this approach to incorporating genotypic data into prediction models has potential clinical utility.

## Supporting Information

Figure S1 **Density plots of risk scores in prediction of CHD with addition of GWAS SNPs to conventional risk factors.** A: Plots for CHD, comprised of fatal or non-fatal MI, angioplasty, coronary artery bypass surgery, angina and/or unspecified ischaemic heart disease as a cause of death; B: Plots for diagnoses limited to fatal or non-fatal MI or coronary intervention (angioplasty or coronary artery bypass surgery). Solid lines represent density curves of risk scores using conventional risk factors, dotted lines represent density curves of risk scores using conventional risk factors and SNPs.
(PDF)

Figure S2 **Regression trees to explain residual variance from models with conventional risk factors in prediction of CHD in the EAS.** A: Regression tree derived for CHD, comprised of fatal or non-fatal MI, angioplasty, coronary artery bypass surgery, angina and/or unspecified ischaemic heart disease as a cause of death; B: Regression tree derived for diagnoses limited to fatal or non-fatal MI or coronary intervention (angioplasty or coronary artery bypass surgery).
(PDF)

Figure S3 **ROC curves derived from SNPs identified by regression trees to explain residual variance from models with conventional risk factors in prediction of CHD in the EAS.** A: ROC curve for CHD, comprised of fatal or non-fatal MI, angioplasty, coronary artery bypass surgery, angina and/or unspecified ischaemic heart disease as a cause of death; B: ROC curve for diagnoses limited to fatal or non-fatal MI or coronary intervention (angioplasty or coronary artery bypass surgery).
(PDF)

Table S1 **SNPs with confirmed associations with CHD used in risk prediction models.**
(PDF)

Table S2 **Additional SNPs Associated with CVD used in regression trees.**
(PDF)

Table S3 **Hazard ratios for conventional risk factors and SNPs in prediction of fatal or non-fatal MI or coronary intervention.**
(PDF)

Table S4 **Hazard ratios for conventional risk factors and SNPs in prediction of coronary heart disease.**
(PDF)

Table S5 **Reclassification of subjects based on a predicted risk of 20%.**
(PDF)

## References

[1] Wilson PWF, D’Agostino RB, Levy D, Belanger AM, Silbershatz H, et al (1998) Prediction of Coronary Heart Disease Using Risk Factor Categories. Circulation 97: 1837–1847. 9603539 10.1161/01.cir.97.18.1837

[2] Eichler K, Puhan MA, Steurer J, Bachmann LM (2007) Prediction of first coronary events with the Framingham score: A systematic review. American Heart Journal 153: 722–731. 17452145 10.1016/j.ahj.2007.02.027

[3] Ridker PM, Brown NJ, Vaughan DE, Harrison DG, Mehta JL (2004) Established and Emerging Plasma Biomarkers in the Prediction of First Atherothrombotic Events. Circulation 109: IV–6-19. 10.1161/01.CIR.0000133444.17867.56 15226246

[4] Martin C, Taylor P, Potts H (2008) Construction of an odds model of coronary heart disease using published information: the Cardiovascular Health Improvement Model (CHIME). BMC Medical Informatics and Decision Making 8: 49. 18976488 10.1186/1472-6947-8-49 PMC2601038

[5] Schunkert H, Erdmann J, Samani NJ (2010) Genetics of myocardial infarction: a progress report. European Heart Journal 31: 918–925. 20219748 10.1093/eurheartj/ehq038

[6] Paynter NP, Chasman DI, Pare G, Buring JE, Cook NR, et al (2010) Association Between a Literature-Based Genetic Risk Score and Cardiovascular Events in Women. JAMA 303: 631–637. 20159871 10.1001/jama.2010.119 PMC2845522

[7] Zdravkovic S, Wienke A, Pedersen NL, de Faire U (2007) Genetic influences on angina pectoris and its impact on coronary heart disease. Eur J Hum Genet 15: 872–877. 17487220 10.1038/sj.ejhg.5201846

[8] Fischer M, Broeckel U, Holmer S, Baessler A, Hengstenberg C, et al (2005) Distinct Heritable Patterns of Angiographic Coronary Artery Disease in Families With Myocardial Infarction. Circulation 111: 855–862. 15710764 10.1161/01.CIR.0000155611.41961.BB

[9] Humphries SE, Drenos F, Ken-Dror G, Talmud PJ (2010) Coronary Heart Disease Risk Prediction in the Era of Genome-Wide Association Studies: Current Status and What the Future Holds. Circulation 121: 2235–2248. 20497987 10.1161/CIRCULATIONAHA.109.914192

[10] Brindle P, Emberson J, Lampe F, Walker M, Whincup P, et al.. (2003) Predictive accuracy of the Framingham coronary risk score in British men: prospective cohort study. BMJ 327: 1267-. 10.1136/bmj.327.7426.1267 PMC286248 14644971

[11] Fowkes FGR, Housley E, Cawood EHH, Macintyre CCA, Ruckley CV, et al (1991) Edinburgh Artery Study: Prevalence of Asymptomatic and Symptomatic Peripheral Arterial Disease in the General Population. Int J Epidemiol 20: 384–392. 1917239 10.1093/ije/20.2.384

[12] Price JF, Lee AJ, Rumley A, Lowe GDO, Fowkes FGR (2001) Lipoprotein (a) and development of intermittent claudication and major cardiovascular events in men and women: The Edinburgh Artery Study. Atherosclerosis 157: 241–249. 11427227 10.1016/s0021-9150(00)00719-x

[13] Peden J, Hopewell J, Saleheen D, Chambers J, Hager J, et al (2011) A genome-wide association study in Europeans and South Asians identifies five new loci for coronary artery disease. Nature Genetics 43: 339–344. 21378988 10.1038/ng.782

[14] Schunkert H, Konig IR, Kathiresan S, Reilly MP, Assimes TL, et al (2011) Large-scale association analysis identifies 13 new susceptibility loci for coronary artery disease. Nature Genetics 43: 4. 10.1038/ng.784 PMC3119261 21378990

[15] R Development Core Team (2010) R: A language and environment for statistical computing. In: Computing RFfS, editor. Vienna, Austria.

[16] Therneau T, Lumley T (2010) survival: Survival analysis R package. R package.

[17] Sing T, Sander O, Beerenwinkel N, Lengauer T (2009) ROCR: Visualizing the performance of scoring classifiers. R package. 10.1093/bioinformatics/bti623 16096348

[18] Harrell FEJ (2010) Hmisc: Harrell Miscellaneous. R package.

[19] Pencina MJ, D’ Agostino RBS, D’ Agostino RBJ, Vasan RS (2008) Evaluating the added predictive ability of a new marker: From area under the ROC curve to reclassification and beyond. Statistics in Medicine 27: 157–172. 17569110 10.1002/sim.2929

[20] Pencina MJ, D’Agostino RB, Steyerberg EW (2011) Extensions of net reclassification improvement calculations to measure usefulness of new biomarkers. Statistics in Medicine 30: 11–21. 21204120 10.1002/sim.4085 PMC3341973

[21] Zhao JH, Hornik K, Ripley B (2010) gap: Genetic analysis package. R package.

[22] Therneau T, Atkinson B, Ripley B (2010) rpart: Recursive Partitioning. R package.

[23] Foulkes AS (2009) Applied Statistical Genetics in R; Gentleman R, Hornik K, Parmigiani G, editors. Amherst: Springer.

[24] Thanassoulis G, Vasan RS (2010) Genetic Cardiovascular Risk Prediction: Will We Get There? Circulation 122: 2323–2334. 21147729 10.1161/CIRCULATIONAHA.109.909309 PMC3075800

[25] NICE (2010) Lipid Modification: Cardiovascular risk assessment and the modification of blood lipids for the primary and secondary prevention of cardiovascular disease. National Institute for Health and Clinical Excellence clinical guideline 67. 21834195

[26] Talmud PJ, Cooper JA, Palmen J, Lovering R, Drenos F, et al (2008) Chromosome 9p21.3 Coronary Heart Disease Locus Genotype and Prospective Risk of CHD in Healthy Middle-Aged Men. Clin Chem 54: 467–474. 18250146 10.1373/clinchem.2007.095489

[27] Morrison AC, Bare LA, Chambless LE, Ellis SG, Malloy M, et al (2007) Prediction of Coronary Heart Disease Risk using a Genetic Risk Score: The Atherosclerosis Risk in Communities Study. Am J Epidemiol 166: 28–35. 17443022 10.1093/aje/kwm060

[28] Bare LAP, Morrison ACP, Rowland CMMS, Shiffman DP, Luke MMMBAP, et al (2007) Five common gene variants identify elevated genetic risk for coronary heart disease. Genetics in Medicine 9: 682–689. 18073581 10.1097/gim.0b013e318156fb62

[29] Kathiresan S, Melander O, Anevski D, Guiducci C, Burtt NP, et al (2008) Polymorphisms Associated with Cholesterol and Risk of Cardiovascular Events. N Engl J Med 358: 1240–1249. 18354102 10.1056/NEJMoa0706728

[30] Zee RYL, Cook NR, Cheng S, Reynolds R, Erlich HA, et al (2004) Polymorphism in the P-selectin and interleukin-4 genes as determinants of stroke: a population-based, prospective genetic analysis. Hum Mol Genet 13: 389–396. 14681304 10.1093/hmg/ddh039

[31] Zee RYL, Cook NR, Cheng S, Erlich HA, Lindpaintner K, et al (2006) Multi-locus candidate gene polymorphisms and risk of myocardial infarction: a population-based, prospective genetic analysis. Journal of Thrombosis and Haemostasis 4: 341–348. 16420563 10.1111/j.1538-7836.2006.01754.x

[32] Humphries SE, Cooper JA, Talmud PJ, Miller GJ (2007) Candidate Gene Genotypes, Along with Conventional Risk Factor Assessment, Improve Estimation of Coronary Heart Disease Risk in Healthy UK Men. Clin Chem 53: 8–16. 17130180 10.1373/clinchem.2006.074591

[33] Davies RW, Dandona S, Stewart AFR, Chen L, Ellis SG, et al (2010) Improved Prediction of Cardiovascular Disease Based on a Panel of Single Nucleotide Polymorphisms Identified Through Genome-Wide Association Studies/Clinical Perspective. Circulation: Cardiovascular Genetics 3: 468–474. 20729558 10.1161/CIRCGENETICS.110.946269 PMC3035486

[34] Ripatti S, Tikkanen E, Orho-Melander M, Havulinna AS, Silander K, et al (2010) A multilocus genetic risk score for coronary heart disease: case-control and prospective cohort analyses. The Lancet 376: 1393–1400. 10.1016/S0140-6736(10)61267-6 PMC2965351 20971364

[35] Hlatky MA, Greenland P, Arnett DK, Ballantyne CM, Criqui MH, et al (2009) Criteria for Evaluation of Novel Markers of Cardiovascular Risk: A Scientific Statement From the American Heart Association. Circulation 119: 2408–2416. 19364974 10.1161/CIRCULATIONAHA.109.192278 PMC2956982

[36] Medina MW, Krauss RM (2009) The Role of HMGCR Alternative Splicing in Statin Efficacy. Trends in Cardiovascular Medicine 19: 173–177. 20005478 10.1016/j.tcm.2009.10.003 PMC2805071

[37] Iles MM (2008) What Can Genome-Wide Association Studies Tell Us about the Genetics of Common Disease? PLoS Genet 4: e33. 18454206 10.1371/journal.pgen.0040033 PMC2323402

