# Supplementary material for: Improvement in Prediction of Coronary Heart Disease Risk over Conventional Risk Factors Using SNPs Identified in Genome-Wide Association Studies
Source: PLoS One. 2013 Feb 27;8(2):e57310. doi: 10.1371/journal.pone.0057310 (PMC3584137; doi:10.1371/journal.pone.0057310)
Supplement: Figure S1 — Density plots of risk scores in prediction of CHD with addition of GWAS SNPs to conventional risk factors. A: Plots for CHD, comprised of fatal or non-fatal MI, angioplasty, coronary artery bypass surgery, angina and/or unspecified ischaemic heart disease as a cause of death; B: Plots for diagnoses limited to fatal or non-fatal MI or coronary intervention (angioplasty or coronary artery bypass surgery). Solid lines represent density curves of risk scores using conventional risk factors, dotted lines represent density curves of risk scores using conventional risk factors and SNPs. (PDF) [file pone.0057310.s001.pdf]

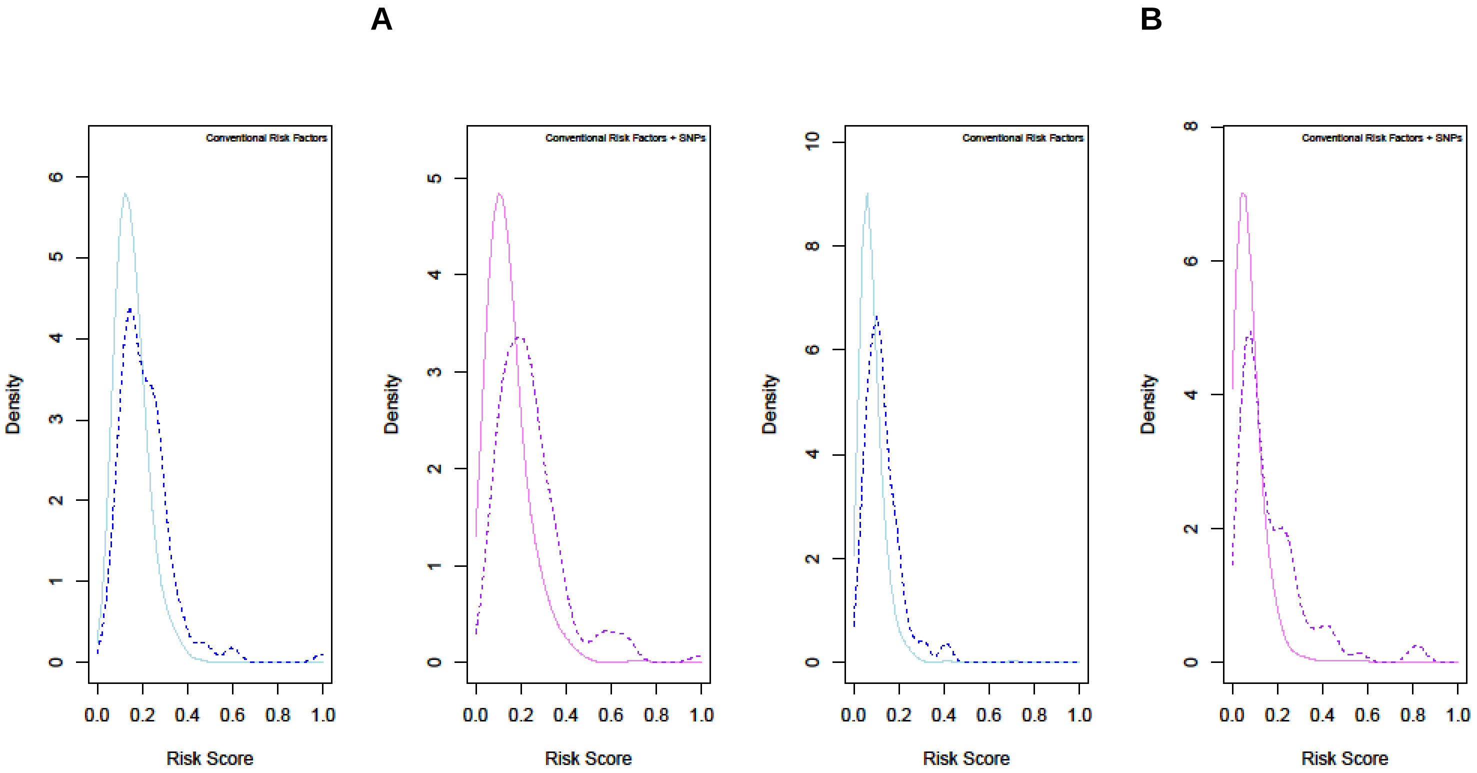

Supplementary Figure S1. Density plots of risk scores in prediction of CHD with addition of GWAS SNPs to conventional risk factors

A: Plots for CHD, comprised of fatal or non-fatal MI, angioplasty, coronary artery bypass surgery, angina and/or unspecified ischaemic heart disease as a cause of death; B: Plots for diagnoses limited to fatal or non-fatal MI or coronary intervention (angioplasty or coronary artery bypass surgery). Solid lines represent density curves of risk scores using conventional risk factors, dotted lines represent density curves of risk scores using conventional risk factors and SNPs.
