# Supplementary material for: Improvement in Prediction of Coronary Heart Disease Risk over Conventional Risk Factors Using SNPs Identified in Genome-Wide Association Studies
Source: PLoS One. 2013 Feb 27;8(2):e57310. doi: 10.1371/journal.pone.0057310 (PMC3584137; doi:10.1371/journal.pone.0057310)
Supplement: Figure S2 — Regression trees to explain residual variance from models with conventional risk factors in prediction of CHD in the EAS. A: Regression tree derived for CHD, comprised of fatal or non-fatal MI, angioplasty, coronary artery bypass surgery, angina and/or unspecified ischaemic heart disease as a cause of death; B: Regression tree derived for diagnoses limited to fatal or non-fatal MI or coronary intervention (angioplasty or coronary artery bypass surgery). (PDF) [file pone.0057310.s002.pdf]

A

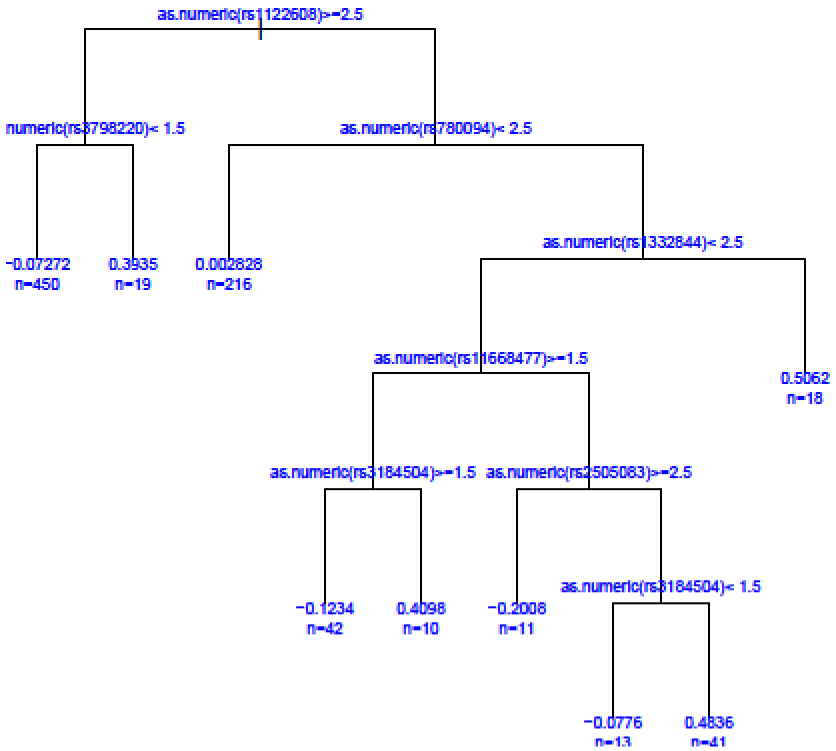
as.numeric(rs1122608)>=2.5
numeric(rs3798220)< 1.5
as.numeric(rs780094)< 2.5
-0.07272
n=450
0.3935
n=19
0.002828
n=216
as.numeric(rs1332844)< 2.5
as.numeric(rs11668477)>=1.5
0.5062
n=18
as.numeric(rs3184504)>=1.5
as.numeric(rs2505083)>=2.5
-0.1234
n=42
0.4098
n=10
-0.2008
n=11
as.numeric(rs3184504)< 1.5
-0.0776
n=13
0.4836
n=41

B

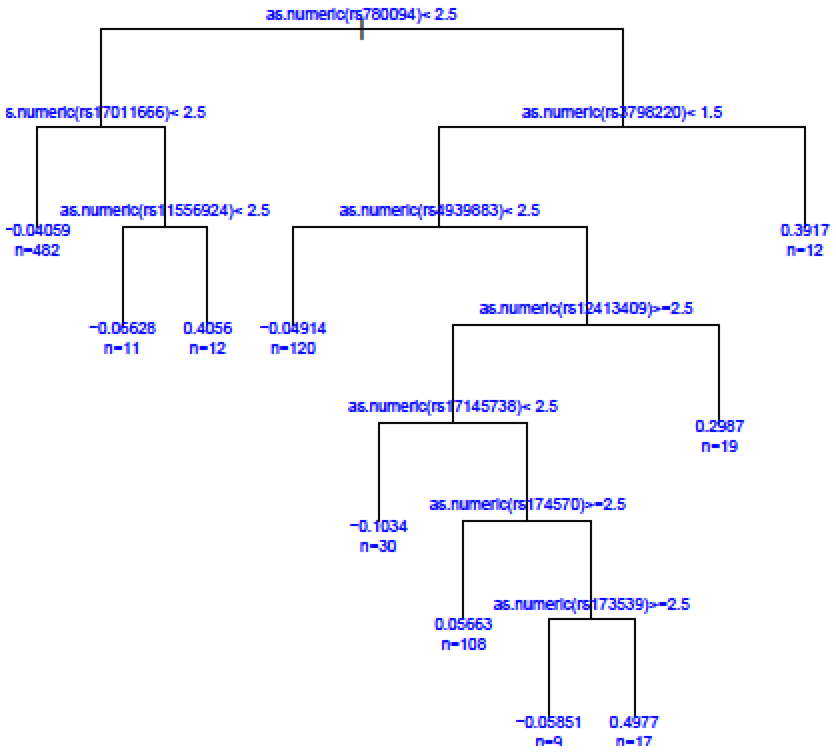

Supplementary Figure S2. Regression trees to explain residual variance from models with conventional risk factors in prediction of CHD in the EAS

A: Regression tree derived for CHD, comprised of fatal or non-fatal MI, angioplasty, coronary artery bypass surgery, angina and/or unspecified ischaemic heart disease as a cause of death; B: Regression tree derived for diagnoses limited to fatal or non-fatal MI or coronary intervention (angioplasty or coronary artery bypass surgery).
