# Supplementary material for: Improvement in Prediction of Coronary Heart Disease Risk over Conventional Risk Factors Using SNPs Identified in Genome-Wide Association Studies
Source: PLoS One. 2013 Feb 27;8(2):e57310. doi: 10.1371/journal.pone.0057310 (PMC3584137; doi:10.1371/journal.pone.0057310)
Supplement: Figure S3 — ROC curves derived from SNPs identified by regression trees to explain residual variance from models with conventional risk factors in prediction of CHD in the EAS. A: ROC curve for CHD, comprised of fatal or non-fatal MI, angioplasty, coronary artery bypass surgery, angina and/or unspecified ischaemic heart disease as a cause of death; B: ROC curve for diagnoses limited to fatal or non-fatal MI or coronary intervention (angioplasty or coronary artery bypass surgery). (PDF) [file pone.0057310.s003.pdf]

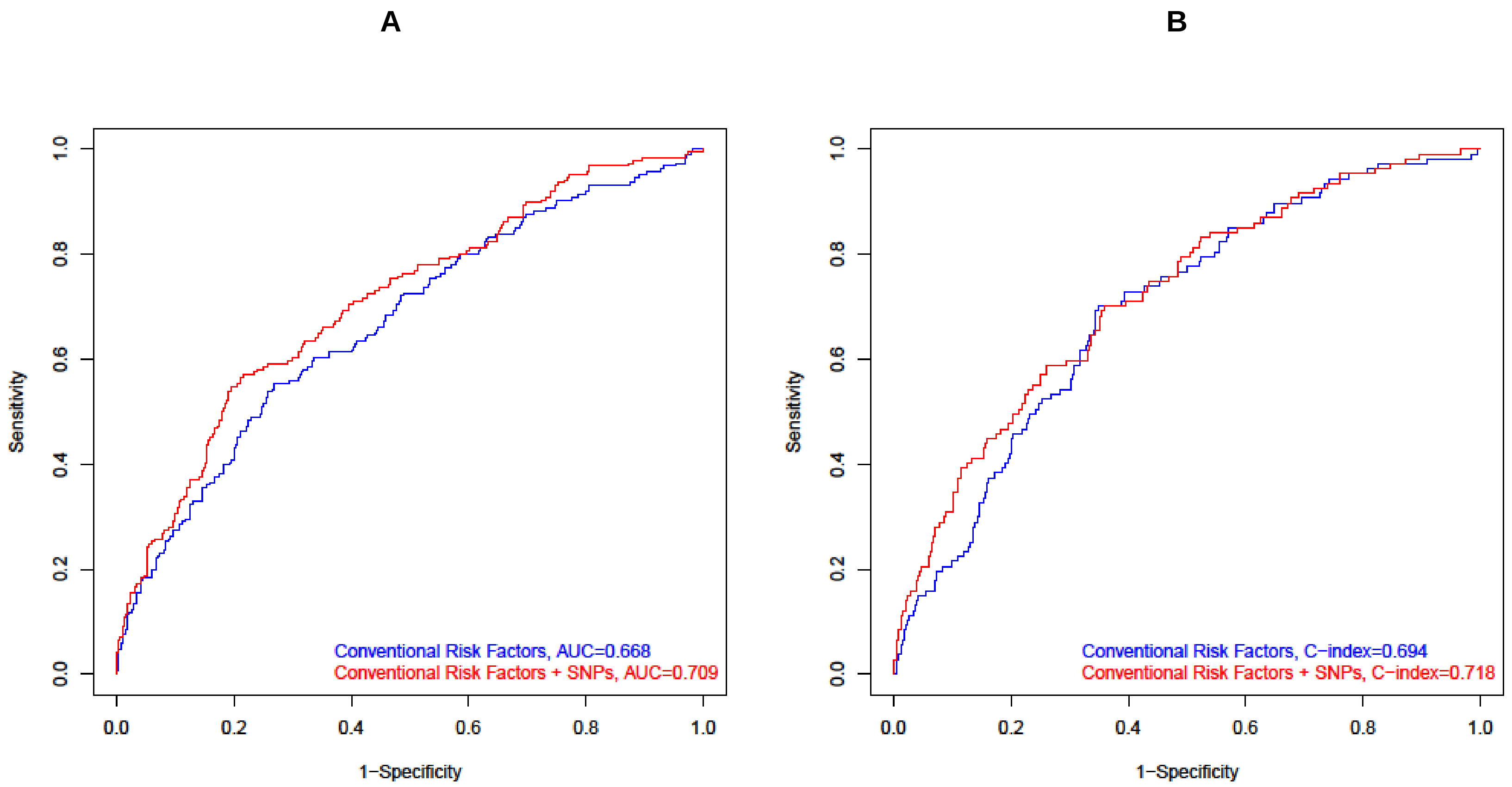

Supplementary Figure S3. ROC curves derived from SNPs identified by regression trees to explain residual variance from models with conventional risk factors in prediction of CHD in the EAS

A: ROC curve for CHD, comprised of fatal or non-fatal MI, angioplasty, coronary artery bypass surgery, angina and/or unspecified ischaemic heart disease as a cause of death; B: ROC curve for diagnoses limited to fatal or non-fatal MI or coronary intervention (angioplasty or coronary artery bypass surgery).
