# Supplementary material for: Improvement in Prediction of Coronary Heart Disease Risk over Conventional Risk Factors Using SNPs Identified in Genome-Wide Association Studies
Source: PLoS One. 2013 Feb 27;8(2):e57310. doi: 10.1371/journal.pone.0057310 (PMC3584137; doi:10.1371/journal.pone.0057310)
Supplement: Table S1 — SNPs with confirmed associations with CHD used in risk prediction models. (PDF) [file pone.0057310.s004.pdf]

Supplementary Table S1. SNPs with confirmed associations with CHD used in risk prediction models

SNP	Chr	Position (b37)	HapMap (CEU)			EAS (Metabochip)				
			Alleles	Minor allele	MAF	Alleles	Minor allele	MAF	Call rate	HWE
rs11206510	1	55,268,627	C/T	C	0.16	A/G	G	0.19	0.987	0.438
rs17114036	1	56,735,409	A/G	G	0.11	A/G	G	0.11	0.924	0.229
rs599839	1	109,623,689	A/G	G	0.28	A/G	G	0.24	0.968	0.642
rs17011666	1	220,865,588	A/G	G	0.17	A/G	G	0.21	0.992	0.196
rs17465637	1	220,890,152	A/C	A	0.27	A/C	A	0.29	0.995	0.403
rs6725887	2	203,454,130	C/T	C	0.16	A/G	G	0.11	0.995	0.861
rs2306374	3	139,602,642	C/T	C	0.18	A/G	G	0.15	0.988	0.916
rs1332844	6	12,996,990	C/T	C	0.39	A/G	G	0.36	0.992	0.452
rs12190287	6	134,256,218	C/G	G	0.40	C/G	C	0.38	0.990	0.956
rs3798220	6	160,881,127	C/T	C	0.00	A/G	G	0.03	0.995	0.933
rs11556924	7	129,450,732	C/T	T	0.39	A/G	A	0.39	0.994	0.937
rs1333049	9	22,115,503	C/G	C	0.46	C/G	G	0.46	0.994	0.790
rs579459	9	135,143,989	C/T	C	0.20	A/G	G	0.19	0.988	0.303
rs2505083	10	30,375,128	C/T	C	0.43	A/G	G	0.42	0.988	0.471
rs1746048	10	44,095,830	C/T	T	0.15	A/G	A	0.14	0.997	0.739
rs12413409	10	104,709,086	A/G	A	0.08	A/G	A	0.07	0.987	0.617
rs974819	11	103,165,777	C/T	T	0.22	A/G	A	0.32	0.993	0.436
rs3184504	12	110,368,991	C/T	T	0.45	A/G	A	0.48	0.984	0.615
rs4773144	13	109,758,713	A/G	G	0.42	A/G	G	0.47	0.855	<0.001
rs2895811	14	99,203,695	C/T	C	0.42	A/G	G	0.41	0.994	0.447
rs3825807	15	76,876,166	A/G	G	0.45	A/G	G	0.49	0.883	0.125
rs4380028	15	76,898,148	C/T	T	0.41	A/G	A	0.42	0.994	0.077
rs12936587	17	17,484,447	A/G	G	0.47	A/G	A	0.46	0.991	0.811
rs1122608	19	11,024,601	G/T	T	0.26	A/C	A	0.24	0.977	0.593
rs2228671	19	11,071,912	C/T	T	0.11	A/G	A	0.12	0.988	0.287
rs9982601	21	34,520,998	C/T	T	0.21	A/G	A	0.14	0.956	0.396
rs7278204	21	34,543,235	A/G	G	0.17	A/G	G	0.15	0.991	0.160
