# Supplementary material for: Improvement in Prediction of Coronary Heart Disease Risk over Conventional Risk Factors Using SNPs Identified in Genome-Wide Association Studies
Source: PLoS One. 2013 Feb 27;8(2):e57310. doi: 10.1371/journal.pone.0057310 (PMC3584137; doi:10.1371/journal.pone.0057310)
Supplement: Table S2 — Additional SNPs Associated with CVD used in regression trees. (PDF) [file pone.0057310.s005.pdf]

Supplementary Table S2. Additional SNPs Associated with CVD used in regression trees

Gene(s)	SNP ID	Chr	Position (b37)	Alleles	MAF	Call Rate	HWE
PCSK9	rs11591147	1	55,278,235	G/T	0.03	100.0	1.000
DOCK7/ANGPTL3	rs10889353	1	62,890,784	A/C	0.34	99.5	0.216
GALNT2	rs4846914	1	228,362,314	A/G	0.43	99.2	0.822
	rs6754295	2	21,059,688	G/T	0.21	100.0	0.558
APOB	rs693	2	21,085,700	A/G	0.49	99.9	0.836
	rs515135	2	21,139,562	C/T	0.23	100.0	0.712
GCKR	rs780094	2	27,594,741	C/T	0.39	98.7	0.878
ABCG5	rs6756629	2	43,918,594	A/G	0.08	99.9	0.026
ABCG8	rs6544713	2	43,927,385	C/T	0.29	99.6	0.480
SDPR	rs10497726	2	192,759,565	A/C	0.23	99.3	0.695
HMGCR	rs12654264	5	74,684,359	A/T	0.40	100.0	0.210
LTA	rs1041981	6	31,648,763	A/C	0.34	100.0	0.719
TNF-a (nr)	rs1800629	6	31,651,010	A/G	0.17	98.7	0.211
LPA	rs6919346	6	160,880,349	C/T	0.19	99.8	0.039
MLXIPL	rs17145738	7	72,620,810	C/T	0.12	98.9	0.049
	rs1801177	8	19,849,988	A/G	0.02	100.0	1.000
LPL	rs326	8	19,863,719	A/G	0.29	100.0	0.863
	rs328	8	19,864,004	C/G	0.12	66.5	0.290
	rs10096633	8	19,875,201	C/T	0.14	99.9	0.634
TRIB1	rs2954029	8	126,560,154	A/T	0.38	100.0	0.537
ABCA1	rs3890182	9	106,687,476	A/G	0.08	99.9	0.490
FADS2	rs174570	11	61,353,788	C/T	0.13	98.9	0.637
FADS1/2	rs1535	11	61,354,548	A/G	0.34	99.9	0.591
	rs12272004	11	116,108,934	A/C	0.04	95.8	0.573
APO AI/C3/A4/A5	rs6589566	11	116,157,633	A/G	0.07	100.0	0.566
	rs662799	11	116,168,917	A/G	0.02	98.9	0.770
COL4A1	rs3742207	13	109,616,599	G/T	0.32	98.9	1.000
LIPC	rs10468017	15	56,465,804	C/T	0.31	95.2	0.061
	rs1532085	15	56,470,658	A/G	0.41	94.5	0.342
	rs173539	16	55,545,545	C/T	0.37	97.7	0.251
CETP	rs3764261	16	55,550,825	A/C	0.34	99.4	0.221
	rs1532624	16	55,562,980	A/C	0.46	99.3	0.891
p22phox	rs4673	16	87,240,737	A/G	0.31	99.5	0.687
SMAD, LIPG	rs4939883	18	45,421,212	C/T	0.19	97.1	0.329
	rs1529729	19	11,024,562	C/T	0.47	98.6	0.401
LDLR	rs11668477	19	11,056,030	A/G	0.18	100.0	0.832
	rs6511720	19	11,063,306	G/T	0.11	99.6	0.616
	rs688	19	11,088,602	C/T	0.42	97.5	0.316
NCAN, CILP2	rs16996148	19	19,519,472	G/T	0.09	99.4	0.785
	rs2304130	19	19,650,528	A/G	0.10	99.8	0.649
	rs157580	19	50,087,106	A/G	0.37	88.8	0.118
APOE	rs2075650	19	50,087,459	A/G	0.16	99.8	0.675
	rs7412	19	50,103,919	C/T	0.28	92.9	0.793
	rs439401	19	50,106,291	C/T	0.38	96.2	0.598
