# Supplementary material for: Improvement in Prediction of Coronary Heart Disease Risk over Conventional Risk Factors Using SNPs Identified in Genome-Wide Association Studies
Source: PLoS One. 2013 Feb 27;8(2):e57310. doi: 10.1371/journal.pone.0057310 (PMC3584137; doi:10.1371/journal.pone.0057310)
Supplement: Table S3 — Hazard ratios for conventional risk factors and SNPs in prediction of fatal or non-fatal MI or coronary intervention. (PDF) [file pone.0057310.s006.pdf]

Supplementary Table S3. Hazard ratios for conventional risk factors and SNPs in prediction of fatal or non-fatal MI or coronary intervention

			Individual CRF HR (95% CI)	Adjusted CRF HR (95% CI)	Individual SNP HR (95% CI)	Adjusted CRF + SNP HR (95% CI)
Age (5 year increase)			1.05 (0.86,1.26)	0.95 (0.78,1.17)		0.95 (0.77,1.17)
Sex = Male			2.26 (1.43,3.57)	2.10 (1.31,3.35)		2.10 (1.29,3.43)
Systolic Blood Pressure (10 mmHg)			1.14 (1.04,1.24)	1.16 (1.05,1.27)		1.16 (1.05,1.28)
Total Cholesterol/HDL Cholesterol			0.72 (0.38,1.35)	0.49 (0.25,0.98)		0.45 (0.22,0.92)
Diabetes = True			1.45 (0.90,2.34)	1.32 (0.81,2.15)		1.28 (0.77,2.13)
Current Smoker = True			1.26 (1.14,1.38)	1.21 (1.09,1.34)		1.27 (1.12,1.43)
Allele*	SNP ID	Gene				
A/**G**	rs11206510	PCSK9			0.82 (0.53,1.26)	0.76 (0.47,1.24)
**A**/C	rs1122608	LDLR			0.91 (0.64,1.29)	0.99 (0.66,1.49)
**A**/G	rs11556924	ZC3HC1			1.08 (0.79,1.49)	1.13 (0.81,1.58)
**C**/G	rs12190287	TCF21			1.03 (0.75,1.43)	1.22 (0.87,1.73)
**A**/G	rs12413409	CYP17A1, CNNM2, NT5C2			0.56 (0.34,0.91)	0.63 (0.38,1.06)
**A**/G	rs12936587	RASD1, SMCR3, PEMT			1.12 (0.83,1.51)	1.13 (0.83,1.55)
A/**G**	rs1332844	PHACTR1			0.97 (0.70,1.34)	1.05 (0.74,1.49)
C/**G**	rs1333049	CDKN2A,			1.02 (0.75,1.39)	1.05 (0.75,1.45)
A/**G**	rs17011666	MIA3			1.07 (0.74,1.55)	0.84 (0.48,1.46)
A/**G**	rs17114036	PPAP2B			0.86 (0.50,1.48)	0.93 (0.51,1.68)
**A**/G	rs1746048	CXCL12			1.06 (0.66,1.72)	0.96 (0.58,1.59)
**A**/C	rs17465637	MIA3			0.87 (0.63,1.20)	0.73 (0.44,1.22)
**A**/G	rs2228671	LDLR			0.75 (0.50,1.13)	0.85 (0.52,1.40)
A/**G**	rs2306374	MRAS			1.37 (0.94,2.00)	1.41 (0.93,2.11)
A/**G**	rs2505083	KIAA1462			1.00 (0.73,1.37)	0.94 (0.67,1.31)
A/**G**	rs2895811	HHIPL1			1.10 (0.81,1.51)	1.24 (0.89,1.72)
**A**/G	rs3184504	SH2B3			1.03 (0.76,1.40)	0.89 (0.64,1.24)
A/**G**	rs3798220	LPA			3.16 (1.38,7.26)	2.24 (0.91,5.49)
A/**G**	rs3825807	ADAMTS7			0.79 (0.58,1.07)	0.80 (0.47,1.37)
**A**/G	rs4380028	ADAMTS7-MORF4L1			1.24 (0.90,1.72)	1.06 (0.60,1.85)
A/**G**	rs4773144	COL4A1, COL4A2			1.03 (0.73,1.47)	1.16 (0.81,1.66)
A/**G**	rs579459	ABO			1.21 (0.82,1.77)	1.06 (0.72,1.57)
A/**G**	rs599839	SORT1			0.99 (0.69,1.44)	0.90 (0.61,1.31)
A/**G**	rs6725887	WDR12			1.01 (0.62,1.65)	1.19 (0.72,1.97)
A/**G**	rs7278204	SLC5A3-MRPS6-KCNE2			0.67 (0.40,1.13)	0.28 (0.11,0.76)
**A**/G	rs974819	PDGFD			0.91 (0.66,1.26)	0.86 (0.60,1.21)
**A**/G	rs9982601	MRPS6			1.08 (0.68,1.71)	0.39 (0.16,0.96)

* Minor allele in bold

* Hazard ratio for allele on right

Note for Supplementary Tables S3 & S4

Hazard ratios were calculated as exp(ß coefficient) from a Cox proportional hazards regression model. The individual CRF and SNP hazard ratios were for each variable independently, without adjustment for any of the other variables (ie. CHD ~ variable). The adjusted CRF were from a model containing all CRF, represented the effect when adjusted for all other CRF (ie. CHD ~ age + sex + SBP + smoking + diabetes and/or glucose intolerance + HDL/total cholesterol). The adjusted CRF + SNP hazard ratios represent the effect after adjustment for all CRF and SNPs (ie. CHD ~ age + sex + SBP + smoking + diabetes and/or glucose intolerance + HDL/total cholesterol + SNP1 + SNP2 + … + SNP27).
