# Supplementary material for: Improvement in Prediction of Coronary Heart Disease Risk over Conventional Risk Factors Using SNPs Identified in Genome-Wide Association Studies
Source: PLoS One. 2013 Feb 27;8(2):e57310. doi: 10.1371/journal.pone.0057310 (PMC3584137; doi:10.1371/journal.pone.0057310)
Supplement: Table S4 — Hazard ratios for conventional risk factors and SNPs in prediction of coronary heart disease. (PDF) [file pone.0057310.s007.pdf]

Supplementary Table S4. Hazard ratios for conventional risk factors and SNPs in prediction of coronary heart disease

			Individual CRF HR (95% CI)	Adjusted CRF HR (95% CI)	Individual SNP HR (95% CI)	Adjusted CRF + SNP HR (95% CI)
Age (5 year increase)			1.03 (0.88,1.19)	0.93 (0.79,1.09)		0.91 (0.77,1.07)
Sex = Male			1.55 (1.10,2.19)	1.48 (1.03,2.13)		1.48 (1.02,2.16)
Systolic Blood Pressure (10 mmHg)			1.15 (1.07,1.23)	1.16 (1.07,1.26)		1.16 (1.07,1.26)
Total Cholesterol/HDL Cholesterol			1.27 (0.83,1.96)	0.98 (0.62,1.55)		1.04 (0.65,1.68)
Diabetes = True			1.43 (0.98,2.09)	1.40 (0.95,2.06)		1.39 (0.92,2.10)
Current Smoker = True			1.29 (1.18,1.41)	1.22 (1.11,1.33)		1.23 (1.11,1.36)
Allele*	SNP ID	Gene				
A/**G**	rs11206510	PCSK9			0.90 (0.65,1.25)	0.84 (0.59,1.21)
**A**/C	rs1122608	LDLR			0.68 (0.52,0.88)	0.70 (0.52,0.94)
**A**/G	rs11556924	ZC3HC1			1.00 (0.78,1.28)	1.02 (0.79,1.31)
**C**/G	rs12190287	TCF21			1.19 (0.92,1.54)	1.25 (0.95,1.64)
**A**/G	rs12413409	CYP17A1, CNNM2, NT5C2			0.74 (0.49,1.14)	0.69 (0.44,1.08)
**A**/G	rs12936587	RASD1, SMCR3, PEMT			1.05 (0.83,1.33)	1.07 (0.83,1.36)
A/**G**	rs1332844	PHACTR1			1.18 (0.92,1.52)	1.27 (0.97,1.65)
C/**G**	rs1333049	CDKN2A,			0.91 (0.71,1.16)	0.94 (0.72,1.23)
A/**G**	rs17011666	MIA3			0.94 (0.70,1.27)	0.82 (0.52,1.28)
A/**G**	rs17114036	PPAP2B			0.73 (0.46,1.16)	0.81 (0.50,1.32)
**A**/G	rs1746048	CXCL12			0.95 (0.65,1.38)	0.86 (0.58,1.27)
**A**/C	rs17465637	MIA3			0.98 (0.75,1.27)	0.83 (0.56,1.22)
**A**/G	rs2228671	LDLR			0.79 (0.57,1.10)	1.01 (0.68,1.49)
A/**G**	rs2306374	MRAS			1.08 (0.78,1.50)	1.03 (0.73,1.45)
A/**G**	rs2505083	KIAA1462			0.96 (0.74,1.23)	0.88 (0.67,1.16)
A/**G**	rs2895811	HHIPL1			1.24 (0.97,1.57)	1.30 (1.00,1.70)
**A**/G	rs3184504	SH2B3			1.02 (0.80,1.29)	1.00 (0.77,1.30)
A/**G**	rs3798220	LPA			2.62 (1.22,5.61)	2.63 (1.11,6.20)
A/**G**	rs3825807	ADAMTS7			0.84 (0.66,1.07)	0.97 (0.63,1.50)
**A**/G	rs4380028	ADAMTS7-MORF4L1			1.24 (0.96,1.60)	1.28 (0.81,2.02)
A/**G**	rs4773144	COL4A1, COL4A2			0.85 (0.64,1.13)	0.96 (0.72,1.28)
A/**G**	rs579459	ABO			1.12 (0.82,1.53)	1.09 (0.79,1.50)
A/**G**	rs599839	SORT1			1.13 (0.86,1.50)	1.08 (0.80,1.44)
A/**G**	rs6725887	WDR12			0.91 (0.61,1.36)	0.98 (0.65,1.47)
A/**G**	rs7278204	SLC5A3-MRPS6-KCNE2			1.02 (0.73,1.44)	0.81 (0.36,1.82)
**A**/G	rs974819	PDGFD			0.99 (0.76,1.27)	1.01 (0.77,1.33)
**A**/G	rs9982601	MRPS6			0.92 (0.65,1.30)	0.81 (0.36,1.83)

* Minor allele in bold

* Hazard ratio for allele on right

Note for Supplementary Tables S3 & S4

Hazard ratios were calculated as exp(ß coefficient) from a Cox proportional hazards regression model. The individual CRF and SNP hazard ratios were for each variable independently, without adjustment for any of the other variables (ie. CHD ~ variable). The adjusted CRF were from a model containing all CRF, represented the effect when adjusted for all other CRF (ie. CHD ~ age + sex + SBP + smoking + diabetes and/or glucose intolerance + HDL/total cholesterol). The adjusted CRF + SNP hazard ratios represent the effect after adjustment for all CRF and SNPs (ie. CHD ~ age + sex + SBP + smoking + diabetes and/or glucose intolerance + HDL/total cholesterol + SNP1 + SNP2 + … + SNP27).
