# Supplementary material for: Improvement in Prediction of Coronary Heart Disease Risk over Conventional Risk Factors Using SNPs Identified in Genome-Wide Association Studies
Source: PLoS One. 2013 Feb 27;8(2):e57310. doi: 10.1371/journal.pone.0057310 (PMC3584137; doi:10.1371/journal.pone.0057310)
Supplement: Table S5 — Reclassification of subjects based on a predicted risk of 20%. (PDF) [file pone.0057310.s008.pdf]

Supplementary Table S5. Reclassification of subjects based on a predicted risk of 20%

	Conventional Risk Factors Alone		Conventional Risk Factors & SNPs	
	No event	Event	No event	Event
**GWAS SIGNIFICANT SNPs**				
**CHD (n=508, 131 incident events)**				
<20%	495	72	491	56
≥20%	14	9	18	25
**Angiographically Confirmed CHD (n=590, 81 incident events)**				
<20%	304	68	306	58
≥20%	73	63	71	73
**REGRESSION TREE SNPs**				
**Severe CHD**				
<20%	546	199	535	184
≥20%	8	15	19	30
**Any CHD**				
<20%	363	86	380	80
≥20%	127	100	110	106

GWAS significant SNPs

- OR of having severe CHD with a predicted risk of 20% or greater based on conventional risk factors was 4.42 (95%CI 1.78,10.46), whereas the odds of having severe CHD with a predicted risk of 20% or greater based on conventional risk factors and GWAS SNPs was 12.18 (95%CI 6.30,24.03).
- OR of having any CHD with a predicted risk of 20% or greater based on conventional risk factors was 3.86 (95%CI 2.52,5.93), whereas the odds of having severe CHD with a predicted risk of 20% or greater based on conventional risk factors and GWAS SNPs was 5.42 (95%CI 3.54,8.38).

Regression tree SNPs

- OR of having severe CHD with a predicted risk of 20% or greater based on conventional risk factors was 5.14 (95%CI 2.20,12.95), whereas the odds of having severe CHD with a predicted risk of 20% or greater based on conventional risk factors and regression tree selected SNPs was 4.59 (95%CI 2.54,8.48).
- OR of having any CHD with a predicted risk of 20% or greater based on conventional risk factors was 3.32 (95%CI 2.34,4.74), whereas the odds of having any CHD with a predicted risk of 20% or greater based on conventional risk factors and regression tree selected SNPs was 4.58 (95%CI 3.20,6.58).
